# Exposure Among Middle and High School Students to Warning Labels on E-Cigarette Packages Before and After an FDA Requirement, 2018–2019

**DOI:** 10.5888/pcd21.220411

**Published:** 2024-03-14

**Authors:** Kimberly Snyder, Sherry T. Liu, Elisabeth A. Donaldson, Teresa Wang, Andrea Gentzke

**Affiliations:** 1 Office of Science, Center for Tobacco Products, US Food and Drug Administration, Silver Spring, Maryland; 2 Office on Smoking and Health, National Center for Chronic Disease Prevention and Health Promotion, Centers for Disease Control and Prevention, Atlanta, Georgia

## Abstract

**Introduction:**

Beginning August 10, 2018, a US Food and Drug Administration (FDA) rule required all e-cigarette packages to have a health warning. We examined exposure among middle and high school students to e-cigarette warnings before and after the compliance date of the FDA’s deeming rule, a rule allowing the FDA to regulate e-cigarettes, cigars, and other products.

**Methods:**

We analyzed data from middle and high school students participating in the 2018 and 2019 National Youth Tobacco Survey. We generated weighted prevalence estimates for any exposure (“rarely,” “sometimes,” “most of the time,” or “always”) and high exposure (“most of the time” or “always”) to warnings. We used independent 2-sided *t* tests to examine differences in exposure between 2018 and 2019 and χ^2^ tests to examine differences in any exposure and high exposure by demographic characteristics and tobacco use behaviors in 2019. Analyses excluded respondents who reported they had not seen an e-cigarette package.

**Results:**

In 2019, 68.0% (vs 67.7% in 2018) of students reported any past 30-day exposure to e-cigarette warning labels and 35.0% (vs 28.7% in 2018) reported high exposure; we observed differences in the proportion of students reporting any and high exposure to warning labels across demographic characteristics and tobacco use behaviors. From 2018 to 2019, report of any and high exposure to e-cigarette warning labels increased among students who currently used any tobacco product and e-cigarettes. We observed increases in high exposure to e-cigarette warning labels overall, and among male students, female students, non-Hispanic White students, and middle and high school students.

**Conclusion:**

After implementation of the health warnings per the FDA’s deeming rule, the percentage of current tobacco users and e-cigarette users among middle and high school students who reported any and high exposure to e-cigarette warning labels increased. Continued monitoring of reactions can inform if warnings are achieving their regulatory goal.

**Summary**

**What is already known on this topic?**

Use of any tobacco product by children and adolescents is not safe. E-cigarette warning labels are one way to increase understanding of the addictiveness of these products among this population.

**What is added by this report?**

This study describes self-reported exposure to e-cigarette warning labels among middle and high school students before and after the compliance date for the US Food and Drug Administration’s warning label requirement.

**What are the implications for public health practice?**

Taken with other data on awareness and processing of warning labels among young people, findings from this study and continued monitoring of reactions can help to determine whether warning labels are achieving their intended regulatory goal.

## Introduction

Use by children or adolescents of any commercial tobacco products, including e-cigarettes, is not safe (1). Most e-cigarettes contain nicotine, which can cause addiction and harm the developing brain (1). Since 2014, e-cigarettes have been the most commonly used commercial tobacco product among young people in the United States (2). In 2018 and 2019, approximately 3.05 and 4.1 million high school students (2018, 20.8%; 2019, 27.5%) and 570,000 and 1.2 million middle school students (2018, 4.9%; 2019, 10.5%) in the US reported past 30-day e-cigarette use (2–4). From 2017 to 2018, just before the August 10, 2018, compliance date for the US Food and Drug Administration’s (FDA’s) warning label requirement, e-cigarette use increased 77.8% (from 11.7% to 20.8%) among high school students and 48.5% (from 3.3% to 4.9%) among middle school students (2). Furthermore, in 2019, 27.5% of high school students and 10.5% of middle school students reported current e-cigarette use (3,4). More recently, in 2022, approximately 2.14 million high school students (14.1% of all US high school students) and 380,000 middle school students (3.3% of all US middle school students) in the US reported past 30-day e-cigarette use (5).

Since 2009, the FDA has had the authority to regulate the manufacturing, distribution, and marketing of tobacco products, including cigarettes, smokeless tobacco, and roll-your-own tobacco. The FDA finalized a rule (the deeming rule), effective August 8, 2016, that allowed the FDA to regulate e-cigarettes, cigars, and all other products that meet the statutory definition of a tobacco product (1,6). On March 15, 2022, the Consolidated Appropriation Act of 2022 (7) updated the term “tobacco product” to include products that contain “nicotine from any source.” At the time this study was conducted, “tobacco products” were limited to products made or derived from tobacco. Beginning August 10, 2018, as part of the rule made in 2016, to help consumers better understand and appreciate the risks and characteristics of tobacco products, manufacturers were required to add the following warning to e-cigarette packages: “WARNING: This product contains nicotine. Nicotine is an addictive chemical” (6). By August 10, 2018, all e-cigarette packages and advertisements were required to have a health warning statement covering at least 30% of the 2 principal display panels of the package and at least 20% of the area of an advertisement (6). One goal of the health warning is to help ensure that consumers, especially children and adolescents, have an understanding of the presence of nicotine and the addictiveness of tobacco products before they might become addicted to them (6).

Cigarette health warning labels can provide important health information to users and nonusers and may prevent young people from initiating smoking (8). Little published literature exists on the effect of noncigarette tobacco health warnings (9–12). Previous research found differences in the perceptions of harm between e-cigarette and waterpipe tobacco among people with a high level of exposure to their respective warning labels (10).

The objective of this study was to describe self-reported past 30-day exposure to warning labels on e-cigarette packages among US middle and high school students before and after the August 10, 2018, compliance date for health warning labels on e-cigarette packages.

## Methods

Data came from 2 years (2018–2019) of the National Youth Tobacco Survey (NYTS), an annual, cross-sectional, school-based, self-administered survey of US public and private school students in grades 6 through 12. NYTS employs a stratified, 3-stage cluster sample design to produce nationally representative samples. Sample sizes and response rates were 20,189 (68.2%) in 2018 and 19,018 (66.3%) in 2019. Data were collected from approximately February through May in each year. The 2018 NYTS was administered as a paper-and-pencil survey; in 2019, the NYTS transitioned to an electronic survey. For this study, we selected the compliance date of the FDA’s health warning rule, August 10, 2018, as the intervention point. This compliance date went into effect between the 2 cycles of data collection. We defined the “before” period as the 2018 survey cycle (before the compliance date) and the “after” period as the 2019 survey cycle (after the compliance date). The survey is administered by the Centers for Disease Control and Prevention (CDC) and the FDA. The 2018 NYTS and 2019 NYTS were reviewed and approved by the Office of Management and Budget and the institutional review boards of the contracted data collectors and CDC.

### Measures

Exposure to e-cigarette warning labels was assessed by the question, “During the past 30 days, how often did you see a warning label on an e-cigarette package?” Response options were “I did not see an e-cigarette package during the past 30 days,” “never,” “rarely,” “sometimes,” “most of the time,” and “always.” Students who responded rarely, sometimes, most of the time, or always were considered to have any exposure to e-cigarette warning labels. Students who responded most of the time or always were considered to have high exposure (9,10).

Current tobacco product use was defined as use of any tobacco products, including cigarettes, e-cigarettes, hookah, cigars, pipe tobacco, smokeless tobacco (chewing tobacco, snuff, or dip; snus; dissolvable tobacco product), and bidis on 1 or more days during the past 30 days. (In this study, the term “tobacco” refers to commercial tobacco products and not to sacred and traditional use of tobacco by some American Indian communities.) Current e-cigarette use was defined as use of e-cigarettes on 1 or more days during the past 30 days. Because people who use e-cigarettes frequently may have an increased exposure to warning labels, we categorized current e-cigarette users into “frequent” (use on ≥20 days in the past 30 days) and “infrequent” users (use on 1 to 19 days in the past 30 days), as done in other studies (2–4).

We restricted analyses to students who responded to the question on how often they saw a warning label on an e-cigarette package in the past 30 days with the following responses: never, rarely, sometimes, most of the time, and always (n = 4,593 in 2018; n = 8,780 in 2019). We excluded students who reported “I did not see an e-cigarette package during the past 30 days” (n = 14,841 in 2018; n = 9,926 in 2019) and students with missing data (n = 1,067 in total for 2018 and 2019). In addition, we performed an ad hoc analysis to help contextualize findings from our main analysis. In this analysis, we examined the percentage of the study sample who reported they saw an e-cigarette package and were exposed to the warning label, saw an e-cigarette package and were not exposed to the warning label, and did not see an e-cigarette package (Appendix).

### Statistical analyses

We generated weighted prevalence estimates and 95% CIs for any exposure and high exposure to e-cigarette warning labels during 2018 and 2019 overall, by student demographic characteristics, current tobacco product use, current e-cigarette use, and frequency of current e-cigarette use. Demographic characteristics were sex (female and male), school level (middle school and high school), and race and ethnicity (Hispanic, non-Hispanic Black, non-Hispanic White, and non-Hispanic Other [American Indian or Alaska Native, Asian, and Native Hawaiian or Other Pacific Islander]).

We used independent 2-sided *t* tests to examine differences in exposure to e-cigarette warning labels between 2018 and 2019 and χ^2^ tests to examine differences in any and high exposure to e-cigarette warnings labels by demographic characteristics and tobacco product use in 2019. For both tests, a *P* value less than .05 was considered significant. We conducted all analyses in SAS-callable SUDAAN version 11.0.3 (RTI International).

## Results

From 2018 to 2019 the proportion of students who reported they did not see an e-cigarette package decreased from 76.5% (95% CI, 75.3%–77.7%) to 53.1% (95% CI, 51.4%–54.7%) (Appendix).

### Any exposure to e-cigarette warning labels

Among students who saw an e-cigarette package in the past 30 days, reporting of any exposure to e-cigarette warning labels did not change from 2018 (67.7%; 95% CI, 65.6%–69.7%) to 2019 (68.0%; 95% CI, 66.1%–69.7%) (Table 1). However, among current tobacco users (2018: 75.7%, 95% CI, 73.1%–78.1%; 2019: 82.0%, 95% CI, 79.9%–84.0%), current e-cigarette users (2018: 77.4%, 95% CI, 74.3%–80.3%; 2019: 83.1%, 95% CI, 80.9%–85.1%), and current frequent e-cigarette users (2018: 81.0%, 95% CI, 75.8%–85.3%; 2019: 89.2%, 95% CI, 86.5%–91.3%), the percentage of students who reported any exposure to e-cigarette warning labels significantly increased from 2018 to 2019. We observed nonsignificant increases among male students, non-Hispanic Black students, non-Hispanic White students, non-Hispanic Other students, middle school students, and high school students. Female students and Hispanic students had nonsignificant decreases.

**Table 1:** Prevalence of Middle and High School Students With Any Exposure to E-Cigarette Warning Labels Among Those Who Saw an E-Cigarette Package in the Past 30 Days, 2018–2019 National Youth Tobacco Survey^a^

Characteristic	Survey in 2018, % (95% CI) (n = 3,051)	Survey in 2019, % (95% CI) (n = 5,884)	2018 vs 2019 *P* value^b^	2019 *P* value^c^
**Overall**	67.7 (65.6–69.7)	68.0 (66.1–69.7)	.83	—
**Sex**				
Female	67.7 (65.1–70.2)	66.3 (64.2–68.4)	.41	.002
Male	67.7 (64.8–70.4)	69.9 (67.7–72.0)	.22	
**Race and ethnicity**				
Hispanic	64.7 (61.1–68.1)	64.3 (61.7–66.7)	.84	<.001
Non-Hispanic Black	51.7 (45.6–57.7)	56.6 (51.9–61.1)	.20	
Non-Hispanic White	72.5 (70.0–74.9)	73.8 (71.7–75.9)	.41	
Non-Hispanic Other	66.5 (61.3–71.2)	68.2 (64.0–72.1)	.60	
**School level**				
Middle school	57.4 (54.1–60.6)	59.5 (56.9–62.0)	.31	<.001
High school	72.6 (70.7–74.4)	74.0 (71.8–76.1)	.35	
**Current any tobacco use (including e-cigarettes)**				
Yes	75.7 (73.1–78.1)	82.0 (79.9–84.0)	<.001	<.001
No	59.9 (57.2–62.6)	59.5 (57.3–61.7)	.82	
**Current e-cigarette use**				
Yes	77.4 (74.3–80.3)	83.1 (80.9–85.1)	.003	<.001
No	60.6 (58.1–63.1)	60.1 (58.0–62.3)	.78	
**Frequency of current e-cigarette use**				
Frequent (≥20 days)	81.0 (75.8–85.3)	89.2 (86.5–91.3)	.003	<.001
Infrequent (1–19 days)	75.7 (72.4–78.7)	79.9 (77.1–82.4)	.04	

a Respondents who indicated seeing an e-cigarette warning label rarely, sometimes, most of the time, or always were considered to have any exposure to e-cigarette warning labels. Analyses were restricted to respondents who reported a response other than “I did not see an e-cigarette package during the past 30 days.” In 2018, n = 4,593 (23.6% of unweighted sample); in 2019, n = 8,780 (46.9% of unweighted sample).

b Independent *t* tests were performed to examine differences between 2018 and 2019;* P* < .05 considered significant.

c Based on χ^2^ test to compare exposure to e-cigarette warning labels by levels of each covariate in 2019; *P* < .05 considered significant.

In 2019, more than three-fourths of students who reported current e-cigarette use (83.1%; 95% CI, 80.9%–85.1%) and current use of any tobacco product (including e-cigarettes) (82.0%; 95% CI, 79.9%–84.0%) reported any exposure to e-cigarette warning labels. Among students who currently used e-cigarettes, any exposure to e-cigarette warning labels was 89.2% (95% CI, 86.5%–91.3%) among frequent users and 79.9% (95% CI, 77.1%–82.4%) among infrequent users (*P* < .001). A higher percentage of male students than female students (69.9%; 95% CI, 67.7%–72.0% vs 66.3%; 95% CI, 64.2%–68.4%; *P* = .002) and high school students than middle school students (74.0%; 95% CI, 71.8%–76.1% vs 59.5%; 95% CI, 56.9%–62.0%, *P* < .001) reported any exposure to e-cigarette warning labels in 2019. By race and ethnicity in 2019, non-Hispanic White students had the highest prevalence (73.8%; 95% CI, 71.7%–75.9%) and non-Hispanic Black students had the lowest prevalence (56.6%; 95% CI, 51.9%–61.1%) of any exposure to e-cigarette warning labels.

### High exposure to e-cigarette warning labels

Among students who had seen an e-cigarette package in the past 30 days, the percentage reporting high exposure to e-cigarette warning labels significantly increased from 28.7% (95% CI, 27.1%–30.5%) in 2018 to 35.0% (95% CI, 33.1%–37.1%) in 2019 (Table 2). From 2018 to 2019, we observed significant increases in high exposure to e-cigarette warning labels among female students (2018: 32.6%, 95% CI, 30.1%–35.2% vs 2019: 36.4%, 95% CI, 33.9%–38.9%), male students (2018: 24.4%, 95% CI, 22.2%–26.6% vs 2019: 33.5%, 95% CI, 31.3%–35.7%), non-Hispanic White students (2018: 32.2%, 95% CI, 29.9%–34.5% vs 2019: 41.9%, 95% CI, 39.4%–44.5%), middle school students (2018: 20.5%, 95% CI, 17.8%–23.4% vs 2019: 24.8%, 95% CI, 22.8%–27.0%), high school students (2018: 32.6%, 95% CI, 30.7%–34.5% vs 2019: 42.1%, 95% CI, 39.6%–44.7%), students who currently used any tobacco product (2018: 37.1%, 95% CI, 34.7%–39.6% vs 2019: 54.0%, 95% CI, 51.4%–56.7%), students who currently used e-cigarettes (2018: 37.0%, 95% CI, 34.2%–39.9% vs 2019: 55.1%, 95% CI, 52.4%–57.9%), students who currently do not use any tobacco product (2018: 20.6%, 95% CI, 18.7%–22.7% vs 2019: 23.6%, 95% CI, 22.0%–25.3%), and students who currently used e-cigarettes frequently (2018: 50.2%, 95% CI, 45.3%–55.1% vs 2019: 72.0%, 95% CI, 68.8%–74.9%) and infrequently (2018: 30.6%, 95% CI, 27.7%–33.6% vs 2019: 46.4%, 95% CI, 43.4%–49.4%).

**Table 2:** Prevalence of Middle and High School Students With High Exposure^a^ to E-Cigarette Warning Labels Among Those Who Saw an E-Cigarette Package in the Past 30 Days, 2018–2019 National Youth Tobacco Survey

Characteristic	Survey in 2018, % (95% CI) (n = 1,281)	Survey in 2019, % (95% CI) (n = 2,959)	2018 vs 2019 *P* value^b^	2019 *P* value^c^
**Overall**	28.7 (27.1–30.5)	35.0 (33.1–37.1)	<.001	—
**Sex**				
Female	32.6 (30.1–35.2)	36.4 (33.9–38.9)	.03	.02
Male	24.4 (22.2–26.6)	33.5 (31.3–35.7)	<.001	
**Race and ethnicity**				
Hispanic	24.7 (21.9–27.8)	28.4 (26.0–30.8)	.06	<.001
Non-Hispanic Black	20.5 (15.7–26.4)	26.8 (23.5–30.4)	.05	
Non-Hispanic White	32.2 (29.9–34.5)	41.9 (39.4–44.5)	<.001	
Non-Hispanic Other	28.4 (24.0–33.4)	32.0 (28.3–36.0)	.24	
**School level**				
Middle school	20.5 (17.8–23.4)	24.8 (22.8–27.0)	.01	<.001
High school	32.6 (30.7–34.5)	42.1 (39.6–44.7)	<.001	
**Current any tobacco use (including e-cigarettes)**				
Yes	37.1 (34.7–39.6)	54.0 (51.4–56.7)	<.001	<.001
No	20.6 (18.7–22.7)	23.6 (22.0–25.3)	.03	
**Current e-cigarette use**				
Yes	37.0 (34.2–39.9)	55.1 (52.4–57.9)	<.001	<.001
No	22.6 (20.6–24.7)	24.7 (23.0–26.5)	.12	
**Frequency of current e-cigarette use**				
Frequent (≥20 days)	50.2 (45.3–55.1)	72.0 (68.8–74.9)	<.001	<.001
Infrequent (1–19 days)	30.6 (27.7–33.6)	46.4 (43.4–49.4)	<.001	

a Respondents who indicated seeing an e-cigarette warning label most of the time or always were considered to have high level of exposure to e-cigarette warning labels. Analyses were restricted to respondents who reported a response other than “I did not see an e-cigarette package during the past 30 days.” In 2018: n = 4,593 (23.6% of unweighted sample); 2019: n = 8,780 (46.9% of unweighted sample).

b Independent *t* tests were performed to examine differences between 2018 and 2019;* P* < .05 considered significant.

c *P* values were based on χ^2^ test comparing exposure to e-cigarette warning labels by levels of each covariate in 2019;* P* < .05 considered significant.

In 2019, more than half of students who reported current e-cigarette use (55.1%; 95% CI, 52.4%–57.9%) and current use of any tobacco product (54.0%; 95% CI, 51.4%–56.7%) reported high exposure to e-cigarette warning labels. Among students who currently used e-cigarettes, 72.0% (95% CI, 68.8%–74.9%) of frequent users and 46.4% (95% CI, 43.4%–49.4%) of infrequent users (*P* < .001) reported high exposure to e-cigarette warning labels. The percentage of female students (36.4%; 95% CI, 33.9%–38.9%) reporting high exposure to e-cigarette warning labels in 2019 was higher than the percentage of male students (33.5%; 95% CI, 31.3%–35.7%) (*P* = .02). A higher percentage of high school students (42.1%; 95% CI, 39.6%–44.7%) than middle school students (24.8%; 95% CI, 22.8%–27.0%) (*P* < .001) reported high exposure to e-cigarette warning labels. In 2019, by race and ethnicity, non-Hispanic White students had the highest prevalence (41.9%; 95% CI, 39.4%–44.5%) and non-Hispanic Black students had the lowest prevalence (26.8%; 95% CI, 23.5%–30.4%) of high exposure to e-cigarette warning labels.

## Discussion

Among all middle and high school students, the percentage who reported any exposure to e-cigarette warning labels almost doubled from 2018 to 2019. Additionally, among all middle and high school students, report of exposure to e-cigarette warning labels increased among all demographic groups assessed, and among students who currently used and did not use tobacco products, students who currently used and did not use e-cigarettes, and students who frequently and infrequently used e-cigarettes. The prevalence of middle and high school students reporting high exposure to e-cigarette warning labels in this study (35.0%), measured less than 1 year after implementation of the FDA’s rule on health warnings, was lower than a previously reported estimate (40.3%) of high exposure to government-required warning labels for smokeless tobacco products measured less than 2 years after implementation of the FDA rule on health warnings (9). Kowitt and colleagues, also using data from the 2019 NYTS, found that 22.3% of middle and high school students reported high exposure to cigar warning labels and 7.0% reported high exposure to waterpipe warning labels; however, these estimates differ from those in our study because they did not exclude students who reported they did not see a tobacco product package (10). Although the ability to make direct comparisons between warning labels of different tobacco products can be limited because of differences in warning label requirements, the methods of studies that measure exposure, and the products themselves, our analysis suggests that rates of high exposure to smokeless tobacco and e-cigarette warning labels in the immediate years after implementation were similar to each other.

The prevalence of exposure to warning labels was higher among students who reported current tobacco use and current e-cigarette use than among students who reported not using tobacco or e-cigarettes. Additionally, a higher proportion of high school students than middle school students reported exposure to e-cigarette warning labels. These findings align with the findings of other studies of exposure to tobacco product warning labels among young people. Those studies found that the proportion of young people reporting exposure to warning labels was higher among survey respondents who used tobacco products than among those who did not use tobacco products (9–11) and higher among older respondents than younger respondents (10,11). These findings suggest that warning labels should be one of several strategies used to educate young people about the harm of tobacco use.

The only significant increase in exposure to warning labels across racial and ethnic groups was among non-Hispanic White high school students. Compared with non-Hispanic Black students, Hispanic students, and non-Hispanic students of other races, a higher proportion of non-Hispanic White students who saw an e-cigarette package in the past 30 days reported any and high exposure to e-cigarette warning labels in 2019. One study examining exposure to cigarette and smokeless tobacco warning labels among US middle and high school students also found that a higher proportion of non-Hispanic White students reported exposure compared with all other racial and ethnic groups (8). Similarly, another study found that among students who currently smoked cigars, non-Hispanic Black students and Hispanic students reported lower odds of high exposure to cigar warning labels than non-Hispanic White students (10). These data can inform tailored strategies to educate young people about the harms of tobacco use according to differences observed by demographic categories.

To our knowledge, this is the first study to examine changes in estimates of exposure to e-cigarette warning labels among young people before and after the FDA required warning labels on e-cigarette packages. We found students who reported they had not seen an e-cigarette package in the past 30 days decreased from 76.5% in 2018 to 53.2% in 2019. This finding is consistent with the rise in e-cigarette use that occurred before and during this period. However, direct attribution of the change in the prevalence of e-cigarette use from 2018 to 2019 to actual increases in the use of e-cigarette products may have been affected by methodologic changes to the 2019 survey, which could also have led to the observed higher estimates of use (3,4). This rise in the prevalence of e-cigarette use is also consistent with the rise in e-cigarette advertising during this time (13,14). Thus, we hypothesize it is likely that both the increase in the prevalence of e-cigarette use among middle and high school students, which presumably resulted in an increase in exposure to e-cigarette packages, and the introduction of the FDA-required e-cigarette warning labels contributed to increases in exposure to e-cigarette warning labels among young people from 2018 to 2019. The increase in exposure to e-cigarette packages is reflected by the decrease in the percentage of students who reported not seeing an e-cigarette package (Appendix). Additionally, before the FDA’s requirement, California required warning labels on e-cigarettes and liquid nicotine (15) and as early as 2014, researchers found many e-cigarette manufacturers included voluntary nicotine content warnings on their products; these warnings varied in content and were displayed in font sizes that were smaller than the font size required by the FDA (16–18). The differences that we observed from 2018 to 2019 in exposure to warning labels may have been smaller than the differences we would have observed had the FDA warning labels been the first warning labels students saw on e-cigarette packages.

### Limitations

Our findings are subject to limitations. First, because NYTS changed from a paper-and-pencil survey to an electronic survey in 2019, the changes we observed in self-reported exposure to warning labels from 2018 to 2019 could be partially attributed to the change in survey mode (19). However, across both years studied, the wording of the question stems that assessed exposure to e-cigarette warning labels did not change, so any effect of the change in survey mode was likely to be small (20). Second, in 2018 and 2019 NYTS did not include measures that assessed exposure to cigarette and smokeless tobacco product warning labels. The cigarette and smokeless tobacco product warning labels currently being used were added to packages in 1984 and 2010, respectively. Without measures that assessed awareness of cigarette and smokeless tobacco warning labels, we were limited in our ability to compare awareness of e-cigarette warning labels (intervention group) with tobacco products that did not have changes in warnings during the same time (a potential control group). Third, the measure assessing exposure to the e-cigarette warning label was not included on NYTS until after the FDA deeming rule was effective (August 2016). Given this, our study period focused on the period before and after the FDA’s compliance date (August 2018) and not the rule effective date (August 2016). If warning labels were added to e-cigarettes between August 2016 and August 2018, then our choice of study period may have resulted in an underestimation of exposure to e-cigarette warning labels in response to the FDA’s health warning requirement. Fourth, data were self-reported and may be subject to response and recall bias. Finally, data were collected only from students who attended public or private schools; thus, findings might not be generalizable to all children or adolescents in the US, such as those who are homeschooled or those who have dropped out of school.

### Conclusion

From 2018 to 2019, the overall proportion of middle and high school students who saw an e-cigarette package and reported any exposure to e-cigarette warning labels did not change, while high exposure to e-cigarette warning labels increased. In 2019, 2 in 3 students reported any exposure to warning labels and 1 in 3 students reported high exposure. Interestingly, in 2019, among students reporting any exposure (n = 5,884), about half reported high exposure (n = 2,959). Warning labels are one approach for increasing understanding of the presence of nicotine in and the addictiveness of e-cigarettes among young people. E-cigarette warning labels complement other critical actions at the national, state, and local levels, including outreach campaigns to educate young people about the dangers associated with using e-cigarettes, such as the FDA’s The Real Cost (21), and other tobacco products. Efforts to educate young people on the harms of commercial tobacco use in coordination with the implementation of comprehensive, proven population-based strategies can prevent and reduce tobacco product use and initiation among this population (1,22). It is important to continue to monitor awareness among young people of e-cigarette warning labels, which can promote their understanding of the addictiveness of these nicotine-containing products. Monitoring awareness by demographic group can inform tailored strategies to educate this population about the harms of tobacco use.

## Appendix

**Appendix:** Table. Prevalence of Middle and High School Students’ Exposure to E-Cigarette Warning Labels in the Past 30 Days, 2018–2019 National Youth Tobacco Survey^a^

Characteristic	Saw e-cigarette package and exposed to warning label			Saw e-cigarette package and were not exposed to warning label			Did not see e-cigarette package		
	2018, % (95% CI) (n = 3,051)	2019, % (95% CI) (n = 5,884)	*P* value^b^	2018, % (95% CI) (n = 1,542)	2019, % (95% CI) (n = 2,896)	*P* value^b^	2018, % (95% CI) (n = 14,841)	2019, % (95% CI) (n = 9,926)	*P* value^b^
**Overall**	15.9 (14.9–17.0)	31.9 (30.5–33.4)	<.001	7.6 (7.1–8.2)	15.0 (14.1–16.0)	<.001	76.5 (75.3–77.7)	53.1 (51.4–54.7)	<.001
**Sex**									
Female	16.5 (15.3–17.7)	32.3 (30.7–33.9)	<.001	7.9 (7.2–8.6)	16.4 (15.2–17.6)	<.001	75.6 (74.2–77.0)	51.4 (49.5–53.3)	<.001
Male	15.1 (13.9–16.4)	31.5 (29.7–33.4)	<.001	7.2 (6.5–8.0)	13.6 (12.6–14.6)	<.001	77.7 (76.2–79.1)	55.0 (53.0–56.8)	<.001
**Race and ethnicity**									
Hispanic	15.8 (14.4–17.3)	32.4 (30.3–34.6)	<.001	8.6 (7.7–9.6)	18.0 (16.7–19.4)	<.001	75.5 (73.9–77.1)	49.6 (47.3–51.8)	<.001
Non-Hispanic Black	9.5 (8.1–11.2)	29.5 (26.8–32.5)	<.001	8.9 (7.6–10.4)	22.7 (19.9–25.7)	<.001	81.5 (79.5–83.4)	47.8 (44.6–51.0)	<.001
Non-Hispanic White	17.8 (16.4–19.3)	33.2 (31.3–35.1)	<.001	6.8 (6.0–7.6)	11.8 (10.7–12.9)	<.001	75.4 (73.5–77.3)	55.0 (52.9–57.2)	<.001
Non-Hispanic Other	14.5 (13.0–16.2)	28.2 (25.5–31.0)	<.001	7.3 (6.0–8.9)	13.1 (11.2–15.4)	<.001	78.1 (75.9–80.2)	58.7 (55.2–62.1)	<.001
**School level**									
Middle school	9.8 (8.8–10.9)	26.0 (24.5–27.6)	<.001	7.3 (6.5–8.1)	17.7 (16.2–19.3)	<.001	82.9 (81.4–84.3)	56.2 (54.0–58.4)	<.001
High school	20.6 (19.3–21.9)	36.6 (34.7–38.5)	<.001	7.8 (7.2–8.4)	12.9 (11.8–14.1)	<.001	71.7 (70.2–73.1)	50.6 (48.5–52.6)	<.001
**Current any tobacco use (including e-cigarettes)**									
Yes	48.6 (46.0–51.2)	63.5 (61.2–65.8)	<.001	15.6 (14.0–17.3)	13.9 (12.4–15.5)	.18	35.8 (33.4–38.2)	22.6 (21.1–24.1)	<.001
No	8.7 (8.0–9.3)	22.6 (21.3–23.9)	<.001	5.8 (5.3–6.3)	15.4 (14.3–16.5)	<.001	85.5 (84.6–86.4)	62.0 (60.4–63.7)	<.001
**Current e-cigarette use**									
Yes	54.8 (52.0–57.7)	66.5 (63.9–68.9)	<.001	16.0 (13.9–18.3)	13.6 (12.0–15.2)	.08	29.2 (27.0–31.5)	20.0 (18.4–21.7)	<.001
No	9.5 (8.8–10.3)	23.2 (22.0–24.6)	<.001	6.2 (5.7–6.7)	15.4 (14.3–16.5)	<.001	84.3 (83.4–85.2)	61.4 (59.6–63.0)	<.001
**Frequency of current e-cigarette use**									
Frequent (≥20 days)	72.6 (67.5–77.1)	80.0 (76.8–82.9)	.01	17.0 (13.1–21.8)	9.7 (7.8–12.1)	.003	10.5 (7.5–14.4)	10.2 (8.2–12.6)	.90
Infrequent (1–19 days)	48.7 (45.9–51.4)	60.5 (57.7–63.3)	<.001	15.6 (13.7–17.8)	15.2 (13.4–17.2)	.78	35.7 (33.3–38.1)	24.3 (22.4–26.2)	<.001

^a^ Exposure to e-cigarette warning labels was assessed by the following question: “During the past 30 days, how often did you see a warning label on an e-cigarette package?” Respondents who indicated rarely, sometimes, most of the time, or always were considered exposed. Respondents who indicated never were considered not exposed. Respondents who chose “I did not see an e-cigarette package during the past 30 days” were categorized as not having seen an e-cigarette package.

^b^ Independent *t* tests performed to examine differences between 2018 and 2019; *P* < .05 considered significant.
